# Unsuccessful intravenous D-mannose treatment in PMM2-CDG

**DOI:** 10.1186/s13023-019-1213-3

**Published:** 2019-10-22

**Authors:** Sarah C. Grünert, Thorsten Marquardt, Ekkehart Lausch, Hans Fuchs, Christian Thiel, Martin Sutter, Anke Schumann, Luciana Hannibal, Ute Spiekerkoetter

**Affiliations:** 1 Department of General Pediatrics, Adolescent Medicine and Neonatology, Faculty of Medicine, Medical Center - University of Freiburg, Mathildenstraße 1, 79106 Freiburg, Germany; 2 Department of General Pediatrics, University Children’s Hospital Münster, Münster, Germany; 3 Center for Child and Adolescent Medicine, Department I, University of Heidelberg, 69120 Heidelberg, Germany; 4 Pharmacy Department, Faculty of Medicine, Medical Center - University of Freiburg, Freiburg, Germany; 5 Laboratory of Clinical Biochemistry and Metabolism, Department of General Pediatrics, Adolescent Medicine and Neonatology, Faculty of Medicine, Medical Center - University of Freiburg, Freiburg, Germany

**Keywords:** PMM2-CDG, Congenital disorder of glycosylation, Mannose

## Abstract

**Background:**

PMM2-CDG (Phosphomannomutase 2 - Congenital disorder of glycosylation-Ia; CDG-Ia) is the most common glycosylation defect, often presenting as a severe multisystem disorder that can be fatal within the first years of life. While mannose treatment has been shown to correct glycosylation in vitro and in vivo in mice, no convincing effects have been observed in short-term treatment trials in single patients so far.

**Results:**

We report on a boy with a severe PMM2-CDG who received a continuous intravenous mannose infusion over a period of 5 months during the first year of life in a dose of 0.8 g/kg/day. N-glycosylation of serum glycoproteins and mannose concentrations in serum were studied regularly. Unfortunately, no biochemical or clinical improvement was observed, and the therapy was terminated at age 9 months.

**Conclusion:**

Postnatal intravenous D-mannose treatment seems to be ineffective in PMM2-CDG.

## Background

PMM2-CDG is usually a severe multisystem disorder caused by mutations in the gene that encodes phosphomannomutase 2 (MIM 212065) [1]. This enzyme catalyzes the cytosolic conversion of mannose-6-phosphate to mannose-1-phosphate, thereby generating a key substrate for *N*-glycan biosynthesis. Deficiency of PMM2 enzymatic activity causes N-linked hypoglycosylation of serum and cellular proteins [2] and marked reduction of total serum mannose compared to controls [3]. The phenotype is broad and comprises hypotonia, developmental delay, failure to thrive, cerebellar atrophy, pericardial effusion, seizures, coagulopathy, hepatopathy, gastrointestinal symptoms, hypothyroidism, esotropia, osteopenia and abnormal subcutaneous fat patterns [1, 3–6]. Severe forms are often fatal within the first years of life. There is currently no cure or approved treatment for PMM2-CDG [2].

Studies in fibroblasts of patients with PMM2-CDG have shown that the incorporation of labelled mannose into proteins was significantly reduced and that the size of the lipid-linked oligosaccharide precursor (LLO) was smaller than in controls. Addition of exogenous mannose to the culture medium in a concentration of 250 μmol/L or higher corrected the hypoglycosylation phenotype in fibroblasts [4]. This correction was transient, since biochemical abnormalities reappeared when mannose was removed. This has been replicated in other in vitro [3, 5, 6] and in vivo models [2, 7]. In two different hypomorphic PMM2-CDG mouse models, feeding mannose to pregnant dams reduced [2] or even prevented embryonic lethality [7], demonstrating for the first time a biological effect of mannose in vivo. Short-term oral mannose treatment of PMM2-CDG patients has failed to correct glycosylation [8–11].

Intravenous mannose therapy was previously applied in one 11-month old PMM2-CDG patient [10]. Continuous intravenous mannose led to a unique change of the isoelectrofocusing pattern of serum sialotransferrins with appearance of two extra bands after 3 weeks of treatment. Mannose treatment had no clinical effect during this short study period.

We report biochemical and clinical findings in a boy with PMM2-CDG who received continuous intravenous mannose treatment for 5 months.

## Methods

D-mannose (Euro OTC Pharma) for intravenous administration was obtained as a sterile filtrated (Millex GP 0,22 μm) and non-pyrogenic (proved by LAL-test) 10% solution (wt/vol) in water for injections. This mannose solution was administered continuously through a central vein catheter for a period of 5 months. The dose was slowly increased from 0.1 g/kg/day to a final dosage of 0.8 g/kg/day within 9 days. Compassionate use of this intravenous trial was approved by an interdisciplinary ethics board meeting (June 2018, including geneticists, metabolic physicians, intensive care physicians and neuropediatricians). Written informed consent for this treatment was obtained from both parents in accordance with rules and regulations for critically ill patients treated at Freiburg University Hospital (06.06.2019). Biochemical response to treatment was initially evaluated weekly for the first 2 months, later once a month. Serum mannose concentrations were analysed by GC/MS. N-glycosylation of serum glycoproteins (transferrin and alpha-1-antitrypsin) was studied by isoelectric focusing (IEF) and HPLC as described [12]. Assessment of the clinical response comprised daily physical examinations as well as regular echocardiography and electroencephalography.

## Case report and results

**Fig. 1:**
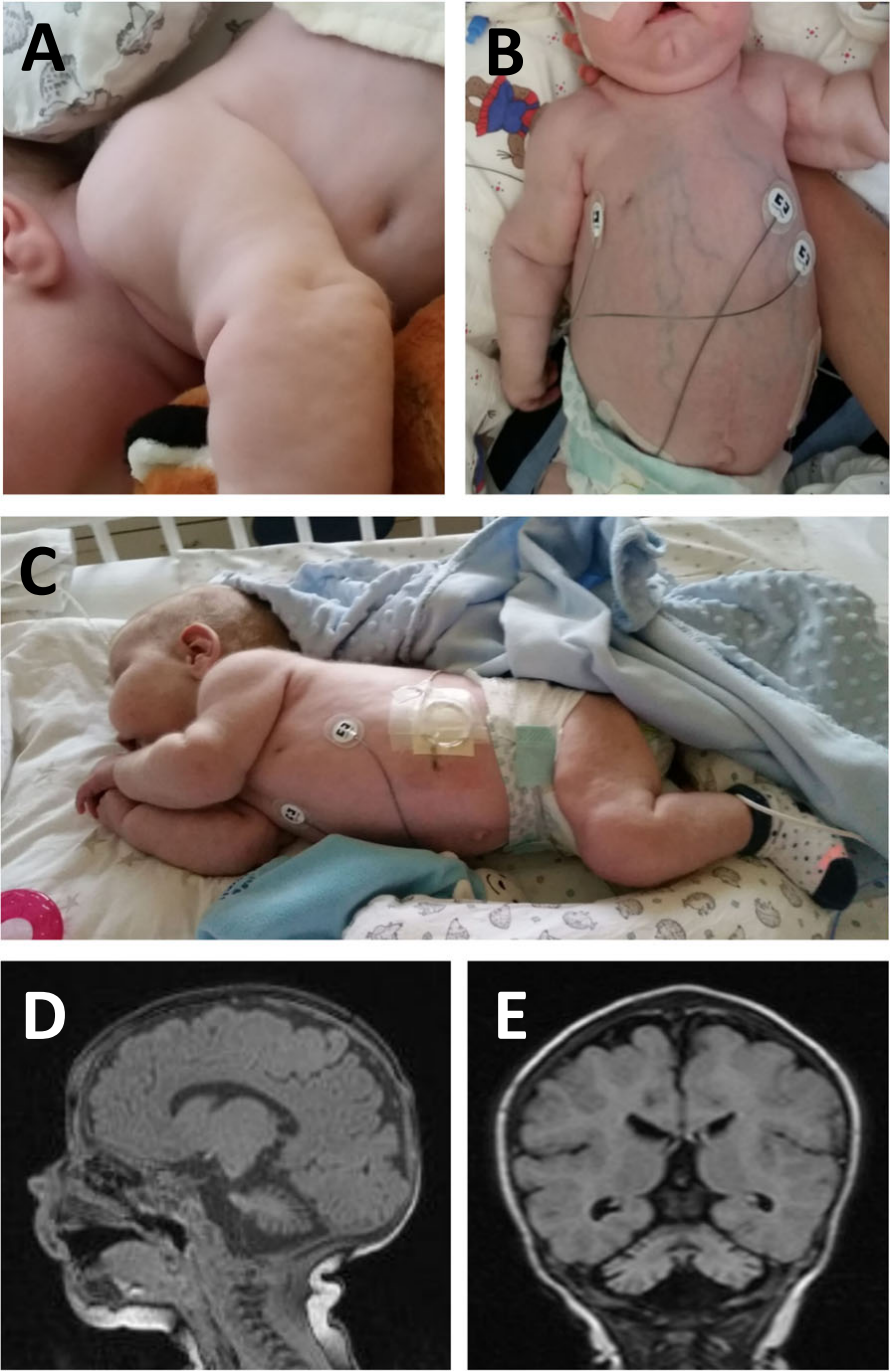
Characteristic clinical findings of PMM2-CDG. **a**-**c** Inverted nipples, abnormal subcutaneous fat distribution, ascites. **d**-**e** Brain MRI of the patient at age 2.5 months showing cerebellar atrophy

The boy is the second child of non-consanguineous Caucasian parents. Birth and perinatal period were unremarkable. At the age of 6 weeks he was admitted to the hospital due to vomiting and diarrhea. He was tachycardic (heart rate 160/min), and a systolic murmur was noted. Echocardiography revealed pericardial effusion, and the child was transferred to our university hospital in poor clinical condition. Additional clinical findings comprised hepatomegaly, inverted nipples, bilateral cryptorchidism and inguinal hernias, muscular hypotonia and an abnormal subcutaneous fat pattern (Fig. 1a-c). Initial laboratory abnormalities included leucocytosis (26.3 G/L), thrombocytosis (720 G/L), anemia (Hb 11.1 g/dl), impaired coagulation parameters (INR 1.19, normal 0.85–1.15; antithrombin III 28%, normal 80–130%), hypomagnesemia (0.53 mmol/L, normal 0.7–0.95 mmol/L), elevated transaminases (ASAT 67 U/L; ALAT 65 U/L, normal 10–50 U/L, respectively), low haptoglobin (10 mg/dL, normal 30–200 mg/dL), severe hypoproteinemia (total protein 2.0 g/dL, normal 5.1–7.3 g/dL; albumin 1.2 g/dL, normal 3.8–5.4 g/dL), proteinuria (protein in spot urine 210 mg/dL, normal < 15 mg/dL; protein/creatinine ratio 3.14 mg/mg, normal < 0.2 mg/mg; albumin in spot urine 171 mg/L, normal < 30 mg/L; albumin/creatinine ratio 255.6 mg/g creatinine, normal < 16.2 mg/g creatinine) and hypothyroidism (TSH 28.1 μU/mL, normal 0.73–8.35 μU/mL; free T4 10.9 pmol/L, normal 11.9–25.6 pmol/L).

Diagnostic work-up revealed serum transferrin and alpha-1-antitrypsin isoelectrofocusing patterns suggestive of CDG-I. PMM2 activity in cultured fibroblasts was markedly reduced (0.1 mU/mg, reference range 1.0–1.5 mU/mg), which associates with moderate phenotypes of the disease [13]. Mutation analysis of the *PMM2* gene yielded compound-heterozygosity for the variants c.422G > A (p.Arg141His) and c.691G > A (p.Val231Met). The mother was heterozygous for the c.691G > A variant, while the father carried the c.422G > A variant. Both sequence changes were reported as pathogenic [14] and impair enzymatic activity [15]. The most common variant p.Arg141His disrupts substrate binding and catalysis [16] and leads to PMM2 proteins with nearly undetectable enzymatic activity [14]. In contrast, variant p.Val231Met retains measurable residual activity in vitro [15] but it is thermally unstable [16]. Mutation of Arg141 to His increases the Km of the PMM2 for substrate D-mannose by one order of magnitude [16].

The pericardial effusion was not hemodynamically relevant, but the patient developed hypertrophic cardiomyopathy with obstruction of the left ventricular outflow tract and was treated with metoprolol. Parenteral nutrition was necessary due to fulminant diarrhea with high protein loss. He also developed ascites requiring permanent drainage and substitution of albumin and antithrombin III. Protein C was severely reduced and substituted as well as fresh frozen plasma. He received erythrocyte and thrombocyte transfusions due to persistent anemia and thrombocytopenia. Brain MRI at the age of 2.5 months showed cerebellar atrophy (Fig. 1d, e), characteristic for PMM2-CDG. At the age of 3 months he developed generalised tonic-clonic seizures that were treated with phenobarbital. The EEG showed a focal epileptic activity over the left parietal hemisphere. Other neurological abnormalities included horizontal nystagmus and esotropia. L-Thyroxin treatment was initiated due to hypothyroidism.

**Fig. 2:**
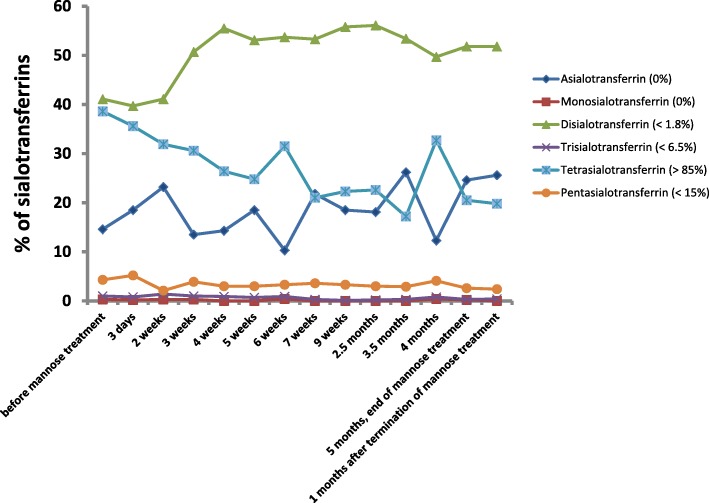
Sialotransferrin pattern of the patient before intravenous mannose therapy, during the treatment trial and after termination of mannose therapy. No relevant change/normalisation of the sialotransferrin pattern could be observed under mannose treatment

At the age of 4 months an intravenous D-mannose trial was started. D-mannose was continuously infused with a starting dose of 0.1 g/kg/d. Over the next 9 days the dose was progressively increased to 0.8 g/kg D-mannose per day. No side effects occurred under this treatment. Analyses of transferrin glycosylation and of serum D-mannose concentrations were performed regularly. The results are shown in Fig. 2. Mannose concentrations in serum before therapy were below 50 μmol/L (*n* = 2). During therapy, mannose concentrations were between 111.2 and 146.7 μmol/L (*n* = 4, mean 128.7 μmol/L) with one higher value (236.3 μmol/L). No major improvement of glycosylation was observed during the 5-month study period. Due to the lack of biochemical and clinical improvement, mannose therapy was terminated at age 9 months. In sum, none of the symptoms (cardiomyopathy, diarrhea and ascites, neurological symptoms) changed significantly during the mannose trial.

At the age of 10 months the patient was dismissed from hospital. His body weight was 9 kg (97th centile), his length 70 cm (7th centile) and his head circumference 44.5 cm (7th centile). During the follow–up he was admitted for albumin infusions and laboratory monitoring once a week. Two short inpatient treatments were necessary due to febrile infections. During one of these episodes *Acinetobacter braumanii* was detected in the ascites and was treated with meropenem.

As the boy had survived several febrile episodes without significant clinical deterioration, the risk of a mild febrile vaccination reaction was considered lower than the risk of severe infections without vaccination despite the known thermolability of the p.Val231Met variant.

At the age of 11 months he was admitted for his regular albumin infusion in good clinical condition. On the same day he received a routine vaccination. On the following day, he was sub-febrile and required oxygen. Few hours later the respiratory situation worsened rapidly, he became tachypnoeic and developed a global respiratory insufficiency. Mechanical ventilation was started and he required up to 100% oxygen. Echocardiography confirmed the pre-existing pericardial effusion and cardiomyopathy, without further deterioration. One day later the blood pressure suddenly dropped (syst. Pressure 20 mmHg) and severe arrhythmias occurred, leading to fatal cardiac failure. No autopsy was performed.

## Discussion

This study aimed to investigate the clinical outcome of a PMM2-CDG boy upon continuous intravenous mannose infusion administered over a period of 5 months within his first year of life (dose of 0.8 g/kg/day). To our knowledge, this is the first study performing a long-term continuous infusion mannose intervention in a severely affected PMM2-CDG infant.

Reports dating back to over two decades described promising effects of mannose administration on PMM2-deficient fibroblasts in vitro [4]. Since then, only a few studies were performed in humans using either enteral (5 patients) or parenteral (1 patient) D-mannose supplementation [8–10]. No clinical or biochemical improvement was observed in any of these short-term treatment studies [8–10]. Because in these trials mannose was only administered for a period of a few weeks, we hypothesized that a longer trial may be helpful to achieve the desired therapeutic effects. Results of long-term studies were not yet available.

Our patient presented with a severe, early diagnosed PMM2-CDG. Mannose treatment was mainly considered due to the lack of other therapeutical options and a poor clinical condition. Although mannose is usually well absorbed and oral administration increases mannose blood levels [17], this therapeutic approach was not possible in our patient due to severe diarrhea and ascites. We could neither observe a clinical nor a biochemical response (including serum transferrin IEF) to D-mannose.

Therapeutic development for patients with PMM2-CDG has suffered from substantial paucity. Table 1 summarizes animal and human studies on the few reported PMM2-CDG treatments. Schneider et al. reported successful prenatal treatment with mannose in a hypomorphic PMM2-CDG mouse model [7]. Embryonic lethality was prevented by feeding mannose to pregnant dams, underlining the essential role of glycosylation in embryonic development [7]. The authors hypothesized that one reason for the unresponsiveness to D-mannose treatment in infancy might be the fact that essential developmental steps during embryogenesis and infancy may have already been irreversibly affected by hypoglycosylation [7].

**Table 1:** Studies on therapy and PMM2-CDG disease modeling with intervention

Description	Subject	Mutation	Outcome	Reference
Continuous infusion of D-mannose (0.8 g/kg/day), for 5 months during the first year of life	Human	c.422G > A (p.Arg141His) and c.691G > A (p.Val231Met).	Unsuccessful	This study
Oral acetazolamide dosages from 6.8 to 16.7 mg/kg per day were well tolerated. Treatment lasted 6-months, followed by a randomized 5-week withdrawal phase.	Human	24 subjects with various mutations	Clinical improvement of cerebellar syndrome	Ann Neurol. 2019;85:740–51.
Five children with PMM2-CDG, received enteral supplementation with d-mannose 100 mg/kg every 3 h for 9 d.	Humans	F119 L/R141H R141H/unknown	Unsuccessful	Acta Paediatr. 1998;87:884–8.
Continuous infusion of mannose 5.7 g/kg/day, for 3 weeks, within first year of life. Stable serum mannose levels up to 2.0 mmol/L were maintained. No signs of liver or renal toxicity.	Humans	Not reported	Unsuccessful	Acta Paediatr. 1997;86:1138–40.
Oral doses of 0.17 g (1.0 mmol) mannose/kg body weight every 3.5 h, for 6 months. Patient was 12 months old at start of treatment.	Human	Phe119Leu/ Arg141His	Unsuccessful	Eur J Pediatr. 1998;157:605–6.
Cultured fibroblasts, 0.25 mmol/L D-mannose added to the culture medium	Human fibroblasts	Not reported	N-linked glycosylation restored to normal	J Clin Invest. 1996;97:1478–87.
Cultured fibroblasts, 1 mmol/L D-mannose added to the culture medium	Human fibroblasts	Not reported	Recovery of GDP-Mannose pools	Glycobiology. 2000;10:829–35.
Cultured fibroblasts, 1 mmol/L D-mannose added to the culture medium In addition, glucose reduction from 5 mmol/L to 0.5 mmol/L improved D-mannose uptake and restoral of N-glycosylation	Human fibroblasts	Not reported	N-linked glycosylation restored to normal	Glycoconj J. 1998;15:499–505.
Cultured fibroblasts, 0.25 mmol/L D-mannose added to the culture medium	Human fibroblasts	Not reported	N-linked glycosylation restored to normal	Biochem Mol Med. 1997;61:161–7.
Treatment of female mice with 9 mg mannose per mL drinking water, 1 week before mating	Mice	Pmm2 R137H/F118 L embryos	Successful	Nat Med. 2011;18:71–3.
Treatment of female mice with 9 mg mannose per mL drinking water, 1 week before mating	Mice	Pmm2 F115 L embryos	Successful	Hum Mol Genet. 2016;25:2182–93.

In accordance with earlier reports, we did not observe any side effects from the high doses of intravenous D-mannose in our patient. In the only other PMM2 patient treated with D-mannose intravenously [10], a dose of up to 5.7 g/kg/day, led to stable serum mannose levels up to 2.0 mmol/l. We administered a significantly lower dose than that reported by Mayatepek et al. such in order to diminish the chance of potential side effects. This led to serum concentrations that remained below the threshold known to correct glycosylation of cultured fibroblasts (see Table 1).

Our patient showed a very rapid clinical deterioration and died shortly after a routine vaccination with a mild febrile reaction. Taking into consideration that the patient carried one null mutation and the sequence variant p.Val231Met known to result in a thermolabile PMM2 protein, it is plausible that the post-vaccination fever was responsible for acute worsening of glycosylation with fatal outcome.

## Conclusion

In conclusion, treatment with intravenous D-mannose over a period of 5 months neither led to a biochemical nor to a clinical response in our patient. Possible reasons for the negative outcome in our patient include: (i) The period of treatment was too short, (ii) Higher doses of mannose may be necessary, (iii) The variant combination in our patient that disrupts both substrate binding and catalysis, as well as protein stability, may be biochemically difficult to correct, even if an adequate concentration of D-mannose does reach the active site of the mutated enzyme.

### Ongoing developments and future outlook in PMM2-CDG therapy

Treatment success may largely depend on a patient’s individual ability to stabilize abnormal PMM2 via cellular chaperones such as has been proposed for Hsp90 [18–20] Along these lines, in silico studies support the exploration of pharmacological chaperones for the stabilization of unstable variants of PMM2 [21]. Two independent studies with mice showed a marked improvement of the embryological lethality of PMM2 hypomorphs upon treatment of mothers with mannose prior to mating, potentially via mechanisms other than rescue of PMM2 enzymatic activity, such as differential gene programming [7].

Development of stem cells of PMM2-CDG patients exhibiting gradual reduction of N-glycosylation will permit the study of PMM2 deficiency at the cellular and molecular levels [22]. In terms of therapeutic strategies, alternative methods have been developed to facilitate the uptake and incorporation of mannose, such as the synthesis of membrane permeable, hydrophobic mannose-1-phosphate-based prodrugs [23, 24]. These hydrophobic mannose-1-phosphate compounds were shown to correct glycosylation in vitro [23] and may represent new therapeutic options. Additionally, a company is currently developing a mannose-1-phoshate formulation using liposomes as the intravenous delivery system [11]. However, this will probably not cross the blood-brain barrier.

Very recently, results of the first clinical trial of acetazolamide in PMM2-CDG (AZATAX) were published [25]. The AZATAX study (*N* = 24 patients) was designed to establish whether acetazolamide, a drug that targets defective CaV2.1 channel activity, could be safely repurposed to treat cerebellar impairment in PMM2-CDG. The rationale was that disrupted N-glycosylation of CaV2.1 contributes to cerebellar syndrome in PMM2-CDG [26], therefore symptoms could be improved upon administration of acetazolamide. Acetazolamide was well tolerated and the majority of patients showed a significant clinical improvement of their cerebellar syndrome [25]. Improvement in prothrombin time, factor X, and antithrombin was also documented [25].

## Data Availability

Not applicable.

## Abbreviations

IEF: Isoelectric focusing
PMM2: Phosphomannomutase
PMM2-CDG: PMM2-congenital disorder of glycosylation
